# Supplementary material for: Monocyte progenitors give rise to multinucleated giant cells
Source: Nat Commun. 2021 Apr 1;12:2027. doi: 10.1038/s41467-021-22103-5 (PMC8016882; doi:10.1038/s41467-021-22103-5)
Supplement: Supplementary file 1 — Supplementary Information [file 41467_2021_22103_MOESM1_ESM.pdf]

Supplementary Information

Monocyte progenitors give rise to multinucleated giant cells

Anne Kathrin Lösslein, Florens Lohrmann, Lisa Scheuermann, Kourosh Gharun, Jana Neuber, Julia Kolter, Aaron James Forde, Christian Kleimeyer, Ying Yee Poh, Matthias Mack, Antigoni Triantafyllopoulou, Micah D. Dunlap, Shabaana A. Khader, Maximilian Seidl, Alexandra Hölscher, Christoph Hölscher, Xue Li Guan, Anca Dorhoi, and Philipp Henneke*

* Correspondence: Philipp Henneke, Philipp.henneke@uniklinik-freiburg.de

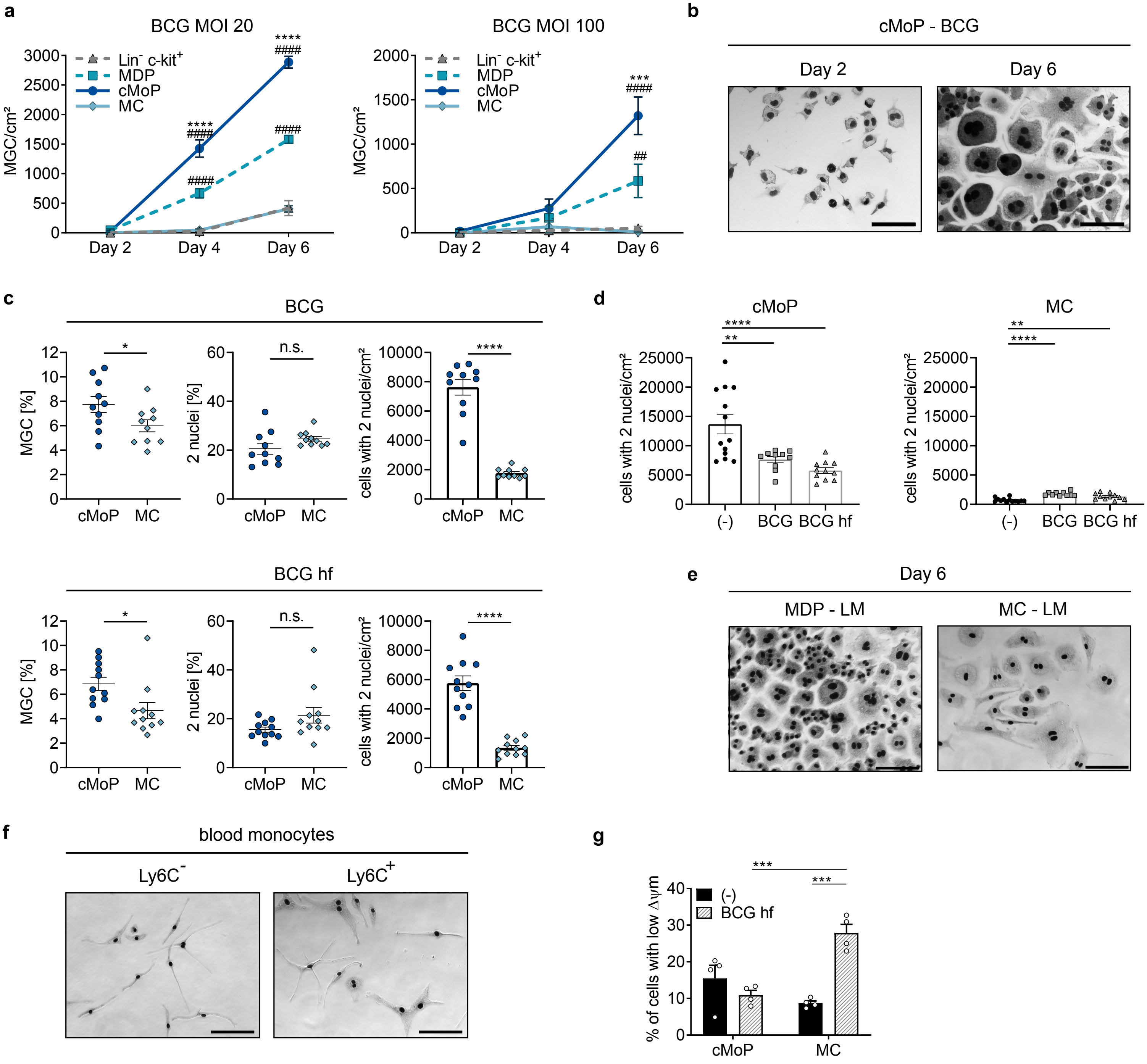

a
BCG MOI 20
BCG MOI 100
Lin- c-kit+
MDP
cMoP
MC
MGC/cm²
Day 2
Day 4
Day 6
b
cMoP - BCG
Day 2
Day 6
c
BCG
BCG hf
MGC [%]
2 nuclei [%]
cells with 2 nuclei/cm²
n.s.
cMoP
MC
d
cMoP
MC
cells with 2 nuclei/cm²
(-)
BCG
BCG hf
e
Day 6
MDP - LM
MC - LM
f
blood monocytes
Ly6C-
Ly6C+
g
(-)
BCG hf
% of cells with low Δψm
cMoP
MC

Supplementary Figure 1, related to Figures 1 and 2: Comparison of bi- and multinucleation during the MGC transformation process

a cMoP (dark blue), MDP (cyan, dashed), Lin^- $c\text{-}kit^+$ cells (grey, dashed) and MC (light blue) were stimulated with BCG (MOI 20 or 100) for 2, 4 and 6 days. Quantification of MGC by Hemacolor staining. Depicted are mean ± SEM of n=3-7 biologically independent samples. Detailed sample numbers (n) for each condition are presented in Supplementary Table 6. ***p=0.001 (cMoP – MDP), ****p< 0.0001 (cMoP – MDP), $^{\#\#}$p=0.0049 (MDP – Lin^- $c\text{-}kit^+$), $^{\#\#}$p=0.0024 (MDP – MC), $^{\#\#\#\#}$p< 0.0001 (refers to MC and Lin^- $c\text{-}kit^+$); (two-way ANOVA, Tukey's multiple comparisons test (MCT)).

b Representative Hemacolor stainings of cMoP from *Cx3cr1*$^{gfp/+}$ mice stimulated with BCG (MOI 20) for 2 (n=5 independent stainings) and 6 days (n=7 independent stainings). Scale bar: 100 µm.

c cMoP (dark blue) and MC (light blue) were stimulated with BCG (MOI 20) or heat-fixed (hf) BCG (10^6/ml) for 6 days. Analysis of MGC formation and binucleated cells after Hemacolor staining. Graphs show mean ± SEM of n=10 (BCG MOI 20) and n=11 (BCG hf) biologically independent samples in 4 experiments. ns= not significant (BCG hf: p=0.0945 and BCG MOI 20: p=0.1127), *p=0.0179 (BCG hf), *p=0.0467 (BCG MOI 20), ****p< 0.0001 (student´s unpaired t-test, two-tailed).

d Formation of binucleated cells of cMoP and MC from *Cx3cr1*$^{gfp/+}$ mice stimulated with BCG (MOI 20) or hf BCG (10^6/ml) for 6 days compared to M-CSF (50 ng/ml) control. Bars show mean ± SEM of n=13 (-), n=10 (BCG MOI 20), n=11 (BCG hf) biologically independent samples in 4 experiments. **p=0.0015 (MC), **p=0.0017 (cMoP), ****p< 0.0001 (one-way ANOVA, Dunnett's MCT).

e Representative Hemacolor stainings of MDP (n=4 independent stainings) and MC (n=6 independent stainings) from *Cx3cr1*$^{gfp/+}$ mice stimulated with LM (4 µg/ml) for 6 days. Scale bar: 100 µm.

f Representative Hemacolor stainings of $Ly6C^+$ and $Ly6C^-$ blood monocytes (n=2 independent stainings) stimulated with LM (4 µg/ml) for 6 days. Scale bar: 100 µm.

g Quantification of mitochondrial membrane potential ($\Delta\Psi m$) by FACS in cMoP and MC stimulated with hf BCG (10^6/ml) compared to control cells (-) after 6 days. Bars show mean ± SEM of n=4 independent biological samples examined over 2 experiments. ***p=0.0009 (cMoP-BCG – MC-BCG), ***p=0.0003 (MC-control – MC-BCG), (two-way ANOVA, Tukey's MCT).

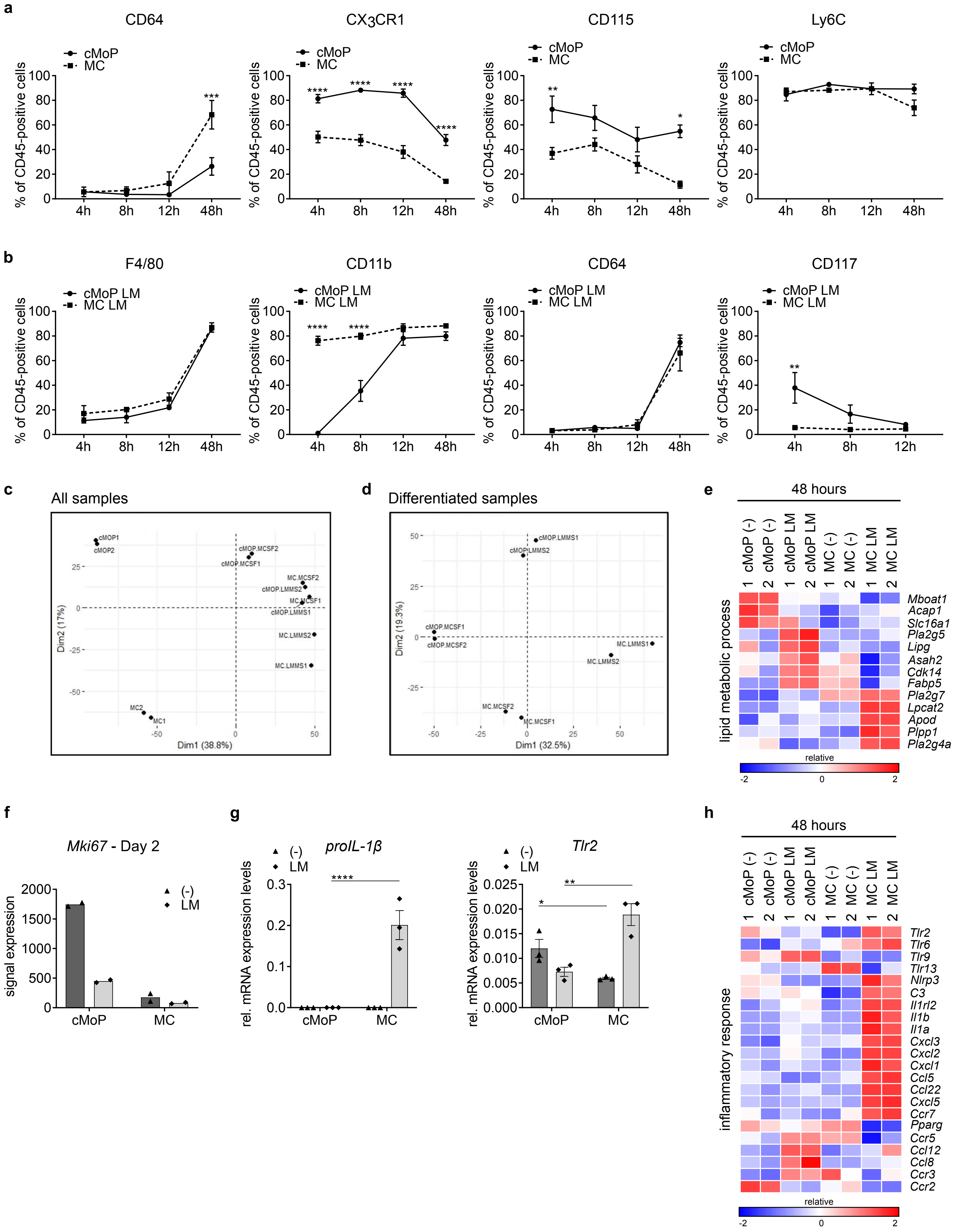
a
CD64
CX3CR1
CD115
Ly6C
cMoP
MC
% of CD45-positive cells
4h
8h
12h
48h
b
F4/80
CD11b
CD64
CD117
cMoP LM
MC LM
c
All samples
Dim1 (38.8%)
Dim2 (17%)
d
Differentiated samples
Dim1 (32.5%)
Dim2 (19.3%)
e
48 hours
lipid metabolic process
Mboat1
Acap1
Slc16a1
Pla2g5
Lipg
Asah2
Cdk14
Fabp5
Pla2g7
Lpcat2
Apod
Plpp1
Pla2g4a
relative
f
Mki67 - Day 2
signal expression
g
proIL-1β
Tlr2
rel. mRNA expression levels
h
48 hours
inflammatory response
Tlr2
Tlr6
Tlr9
Tlr13
Nlrp3
C3
Il1rl2
Il1b
Il1a
Cxcl3
Cxcl2
Cxcl1
Ccl5
Ccl22
Cxcl5
Ccr7
Pparg
Ccr5
Ccl12
Ccl8
Ccr3
Ccr2
relative

Supplementary Figure 2, related to Figure 3: Further analysis of gene expression patterns in cMoP and MC after stimulation with mycobacterial glycolipids

a and b cMoP and MC from *Cx3cr1*$^{gfp/+}$ mice were harvested after 4, 8, 12 or 48 hours in culture with M-CSF (50 ng/ml) alone (a) or after additional stimulation with LM (4 µg/ml) (b) and analyzed for surface marker expression related to CD45^{+} cells by FACS. Depicted are mean ± SEM of n=3-9 biologically independent samples. Detailed sample numbers (n) for each condition are presented in Supplementary Table 4. *p=0.0142, **p=0.0085 (CD115), **p=0.0054 (CD117), ***p=0.0007, ****p< 0.0001 (two-way ANOVA, Sidak's MCT).

c and d Principal component analysis for all samples (c) and cultivated and differentiated samples only (d). Plotted are loadings for principal components PC1 and PC2.

e Heatmap of gene expression in cMoP and MC from *Cx3cr1*$^{gfp/+}$ mice stimulated with LM (4 µg/ml) for 48 hours. Depicted are standardized log2 differences of significantly up- (red) or downregulated (blue) genes in cMoP LM compared to MC LM or cMoP (-) compared to MC (-). Analysis was performed by GO-Term (GO:0006629 lipid metabolic process). If GO-Terms overlap with genes shown in heatmaps in Fig. 3d respective genes are not depicted here again. Gene array was performed with 2 independent biological samples per group (1 = samples 1; 2 = sample 2).

f Bars show mean of *Mki67* expression for cMoP and MC stimulated with LM (4 µg/ml, light grey, diamonds) compared to M-CSF (50 ng/ml) controls (dark grey, triangles) from gene array analysis with n=2 independent biological samples per group.

g qRT-PCR analysis of *Tlr2* and *proIL-1β* mRNA relative to *Gapdh* mRNA in cMoP and MC stimulated with LM (12 µg/ml, light grey, diamonds) for 48 hours compared to M-CSF (50 ng/ml) controls (dark grey, triangles). Bars show mean ± SEM of n=3 biologically independent samples. *p=0.0444, **p=0.0013, ****p< 0.0001 (two-way ANOVA, Sidak's MCT).

h Heatmap of gene expression in cMoP and MC from *Cx3cr1*$^{gfp/+}$ mice stimulated with LM (4 µg/ml) and M-CSF (50 ng/ml) or with M-CSF alone (-) for 48 hours. Shown are standardized log2 differences of representative genes for inflammatory response (GO:0006954). Gene array was performed with 2 independent biological samples per group (1 = sample 1; 2 = sample 2).

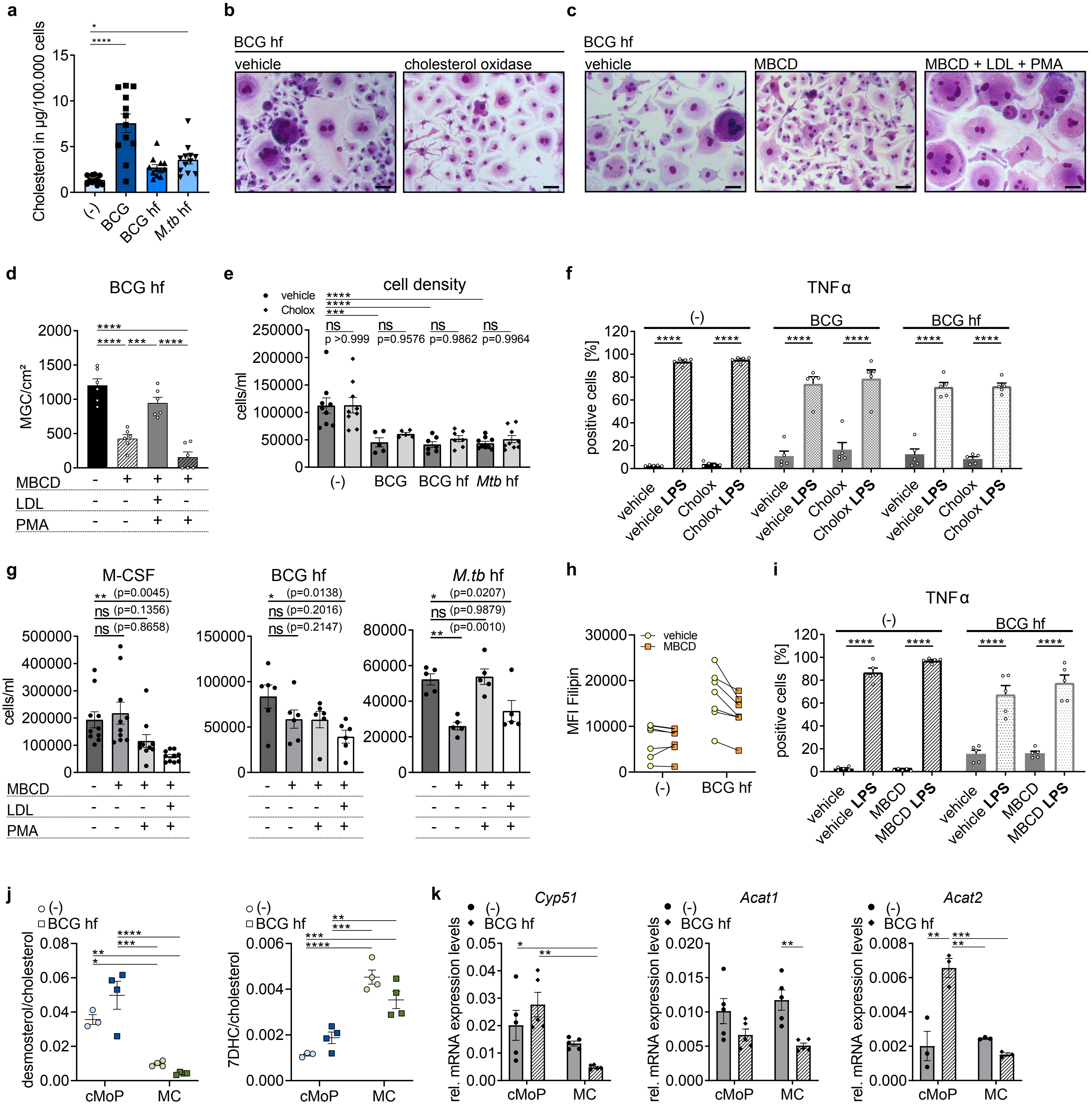
a
Cholesterol in µg/100.000 cells
(-)
BCG
BCG hf
M.tb hf
b
BCG hf
vehicle
cholesterol oxidase
c
BCG hf
vehicle
MBCD
MBCD + LDL + PMA
d
BCG hf
MGC/cm²
MBCD
LDL
PMA
e
cell density
vehicle
Cholox
cells/ml
p >0.999
p=0.9576
p=0.9862
p=0.9964
Mtb hf
f
TNFα
positive cells [%]
vehicle
vehicle LPS
Cholox
Cholox LPS
g
M-CSF
(p=0.0045)
(p=0.1356)
(p=0.8658)
(p=0.0138)
(p=0.2016)
(p=0.2147)
(p=0.0207)
(p=0.9879)
(p=0.0010)
h
MFI Filipin
i
MBCD LPS
j
desmosterol/cholesterol
7DHC/cholesterol
cMoP
MC
k
Cyp51
Acat1
Acat2
rel. mRNA expression levels

Supplementary Figure 3, related to Figures 4 and 5: Complementary data regarding the modification of MGC formation by altering lipid metabolism in cMoP

a Cholesterol Amplex Red Assay of cMoP stimulated for 6 days with BCG (MOI 20), heat-fixed (hf) BCG (10^6/ml) or hf *M.tb* (10 µg/ml) compared to M-CSF control (50 ng/ml). Cholesterol content was normalized to cell number. Bars show mean ± SEM of n=12 biologically independent samples in 4 experiments. *p=0.0279, ****p< 0.0001 (ordinary one-way ANOVA, Dunnett's MCT).

b Representative Hemacolor staining of cMoP from *Cx3cr1*$^{gfp/+}$ mice stimulated with heat-fixed BCG (10^6/ml) and additionally treated with cholesterol oxidase (2 mU/ml) or PBS (vehicle) for 6 days. Scale bar: 50 µm. Gamma-correction was adjusted (0.45) in both images.

c Representative Hemacolor stainings of cMoP from *Cx3cr1*$^{gfp/+}$ mice stimulated with heat-fixed BCG (10^6/ml) and treated with methyl-β-cyclodextrin (MBCD, 1 mmol), LDL (50 µg/ml) or PMA (1 µg/ml) for 6 days. Vehicle control for MBCD was dH_2O. Scale bar: 50 µm. Gamma-correction was adjusted (0.45) in all images.

d Analysis of MGC formation of cMoP from *Cx3cr1*$^{gfp/+}$ mice treated as indicated in Supplementary Fig. 3c. Bars show mean ± SEM of n=6 biologically independent samples examined over 3 experiments. ***p=0.001, ****p< 0.0001 (one-way ANOVA, Tukey's MCT).

e Cell density of cMoP from *Cx3cr1*$^{gfp/+}$ mice stimulated with BCG (MOI 20), heat-fixed BCG (10^6/ml) or heat-fixed *M.tb* (10 µg/ml) and treated with cholesterol oxidase (2 mU/ml, light grey) or equal volume of PBS (vehicle, dark grey) for 6 days determined by FACS counting particles. Bars show mean ± SEM of n=9 for control, n=5 for BCG (MOI 20), n=7 for hf BCG and n=9 for hf *M.tb* biologically independent samples in 2-4 experiments. ns= not significant, ***p=0.0002, ****p< 0.0001 (one-way ANOVA, Sidak's MCT).

f TNFα production of cMoP from *Cx3cr1*$^{gfp/+}$ mice treated as indicated in Supplementary Fig. 3e. LPS (100 ng/ml) was added 5 hours prior to analysis. Bars show mean ± SEM of n=5 independent biological samples examined over 3 experiments. ****p< 0.0001 (one-way ANOVA, Sidak's MCT).

g cMoP from *Cx3cr1*$^{gfp/+}$ mice were stimulated with heat-fixed (hf) BCG (10^6/ml) or hf *M.tb* (10 µg/ml) and treated as indicated in Supplementary Fig. 3c. Cell density was determined by FACS counting particles. Bars show mean ± SEM of n=10 for control (M-CSF), n=6 for hf BCG and n=5 for hf *M.tb* biologically independent samples examined over at least 2 experiments. ns= not significant, *p< 0.05, **p< 0.01 (one-way ANOVA, Dunnett's MCT).

h Filipin staining of sorted cMoP stimulated with heat-fixed BCG (10^6/ml) and additionally treated with methyl-β-cyclodextrin (MBCD, 1 mmol) by FACS. Depicted is the mean fluorescence intensity (MFI) of n=6 biologically independent samples examined over 3 experiments.

i TNFα production of cMoP from *Cx3cr1*$^{gfp/+}$ mice stimulated with heat-fixed BCG (10^6/ml) and additionally treated with methyl-β-cyclodextrin (MBCD, 1 mmol) or equal volume of dH_2O (vehicle) for 6 days. LPS (100 ng/ml) was added 5 hours prior to analysis. Bars show mean ± SEM of n=5 biologically independent samples in 2 experiments. ****p< 0.0001 (one-way ANOVA, Sidak's MCT).

j Desmosterol and 7-dehydrocholesterol (7-DHC) content of cMoP (blue) and MC (green) relative to cholesterol content from sterol analysis described in Fig. 5e. Depicted are mean ± SEM of n=4 independent biological samples (cMoP control with n=3 as one sample could not be analyzed for technical reasons). *p=0.0124, **p=0.0033 (desmosterol), **p=0.0085 (7-DHC), ***p=0.0002 (desmosterol), ***p=0.0002 (7-DHC, cMoP-BCG – MC(-)), ***p=0.001 (7-DHC, cMoP(-) – MC-BCG), ****p< 0.0001 (two-way ANOVA, Tukey's MCT).

k qRT-PCR analysis of *Cyp51, Acat1* and *Acat2* mRNA levels relative to *Gapdh* mRNA of cMoP and MC stimulated with heat-fixed BCG (10^6/ml, diamonds, hatched bars) for 6 days compared to M-CSF (50 ng/ml) controls (circles, grey bars). Bars show mean ± SEM of n=5 (*Cyp51*, *Acat1*) and n=3 (*Acat2*) biologically independent samples in 2 experiments. *p=0.0354, **p=0.0017 (*Cyp51*), **p=0.0093 (*Acat1*), **p=0.0011 (*Acat2*, cMoP(-) – cMoP-BCG), **p=0.0022 (*Acat2*, MC(-) – cMoP-BCG), ***p=0.0005 (two-way ANOVA, Tukey's MCT).

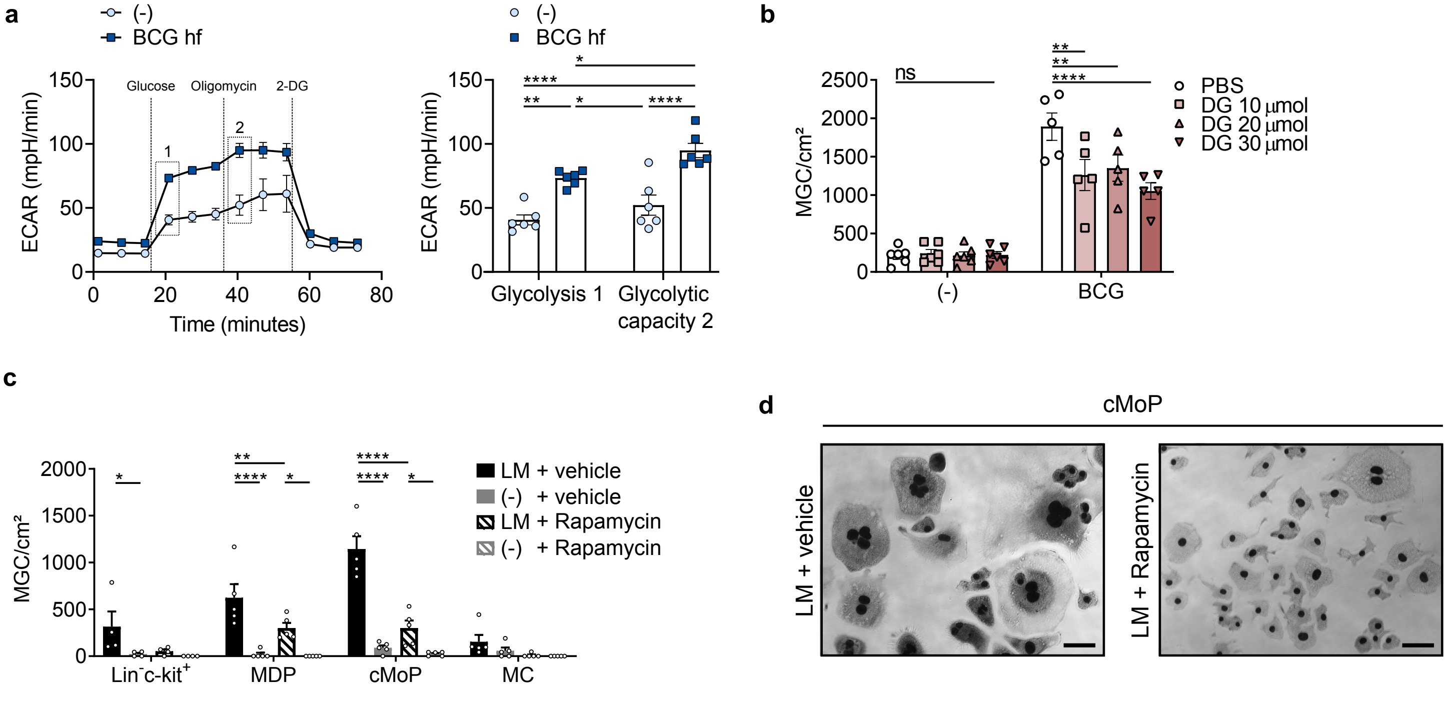

Supplementary Figure 4, related to Figures 4 and 5: Increased glycolysis is relevant for the metabolism of multinucleated giant cells

a Glycolysis Stress Test from cMoP stimulated with heat-fixed (hf) BCG (10^6/ml, dark blue, squares) compared to M-CSF (50 ng/ml) control (light blue, circles). Depicted are extracellular acidification rate (ECAR) over time and ECAR at indicated time points (1 and 2) in bar graphs. Shown are mean ± SEM of n=6 biologically independent samples in 2 experiments. *p=0.0488 (BCG1 - control2), *p=0.0444 (BCG1 - BCG2), **p=0.0017, ****p< 0.0001 (two-way ANOVA, Tukey's MCT).

b cMoP and MC from *Cx3cr1$^{gfp/+}$* mice were stimulated with BCG (MOI 20) and additionally treated with 2-Deoxy-D-glucose (DG; 10 µmol, 20 µmol or 30 µmol) or PBS (vehicle, equal volume as used for 30 µmol DG) for 6 days. MGC were quantified by Hemacolor staining. Bars show mean ± SEM of n=6 (-) and n=5 (BCG) biologically independent samples. ns= not significant (>0.99 for all DG concentrations compared to PBS), **p=0.0017 (10 µmol), **p=0.0074 (20 µmol), ****p< 0.0001 (two-way ANOVA, Dunnett's MCT).

c and d Bone marrow progenitors from *Cx3cr1$^{gfp/+}$* mice were stimulated with LM (4 µg/ml) and additionally treated with rapamycin (10 nmol) or DMSO (vehicle, equivalent amount for 10 nmol rapamycin) for 6 days. MGC were quantified by Hemacolor staining. Representative pictures are shown (scale bar: 50 µm). Bars show mean ± SEM of n=5 independent biological samples (n=4 for Lin^- c-kit^+ cells, as one sample was removed for technical reasons). *p=0.0482 (Lin^- c-kit^+), *p=0.0178 (MDP), *p=0.0339 (cMoP), **p=0.0092, ****p< 0.0001 (two-way ANOVA, Tukey's MCT).

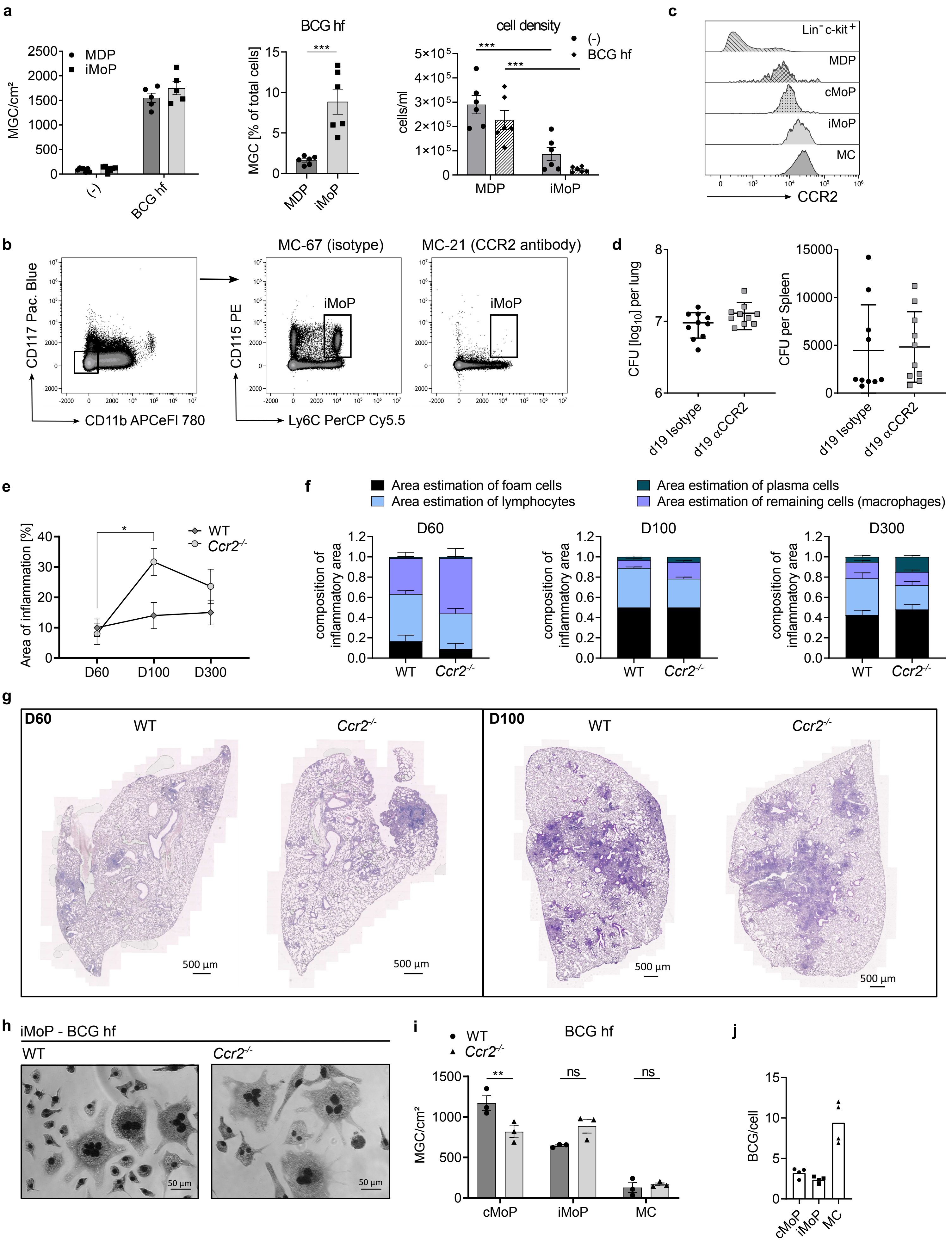
a
MGC/cm²
MDP
iMoP
(-)
BCG hf
BCG hf
MGC [% of total cells]
***
MDP
iMoP
cell density
(-)
BCG hf
cells/ml
***
***
MDP
iMoP
c
Lin⁻ c-kit⁺
MDP
cMoP
iMoP
MC
CCR2
b
CD117 Pac. Blue
CD11b APCeFl 780
MC-67 (isotype)
MC-21 (CCR2 antibody)
CD115 PE
Ly6C PerCP Cy5.5
iMoP
iMoP
d
CFU [log10] per lung
CFU per Spleen
d19 Isotype
d19 αCCR2
d19 Isotype
d19 αCCR2
e
Area of inflammation [%]
WT
Ccr2-/-
*
D60
D100
D300
f
Area estimation of foam cells
Area estimation of lymphocytes
Area estimation of plasma cells
Area estimation of remaining cells (macrophages)
composition of inflammatory area
D60
D100
D300
WT
Ccr2-/-
g
D60
WT
Ccr2-/-
D100
WT
Ccr2-/-
500 µm
h
iMoP - BCG hf
WT
Ccr2-/-
50 µm
i
WT
Ccr2-/-
BCG hf
MGC/cm²
**
ns
ns
cMoP
iMoP
MC
j
BCG/cell
cMoP
iMoP
MC

Supplementary Figure 5, related to Figures 6 and 7: Analysis of the role of CCR2 for progenitor recruitment and giant cell formation

a Comparison of iMoP and MDP cultured for 6 days with heat-fixed (hf) BCG (10^6/ml) and M-CSF (50 ng/ml) or with M-CSF (50 ng/ml) alone (-) with respect to MGC formation and cell density. Depicted are mean ± SEM of n=7 (control MGC/cm²), n=5 (BCG MGC/cm²), n=6 (percentage of total cells and cell density) independent biological samples in at least 2 experiments. ***p=0.0009 (percentage of total cells), (student´s unpaired t-test, two-tailed); ***p=0.0007 (cell density), (two-way ANOVA, Tukey's MCT).

b Representative FACS dot plots of iMoP frequency in the blood at day 19 after aerosol infection with *M.tb* (200 CFU). Mice were injected i.p. daily with 20 µg of a CCR2 specific antibody (MC-21) or isotype control (MC-67) from day 14 to 19.

c CCR2 (MC-21) staining of bone marrow precursor subsets.

d Bacterial loads in lung and spleen at day 19 in the infection model described in Supplementary Fig. 5b. Depicted are mean ± SD of n=10 mice per group examined over 2 experiments.

e C57BL/6 (WT) and *Ccr2*$^{-/-}$ mice were infected with *M.tb* (100 CFU, strain HN878) via aerosol and the lungs analyzed for inflammatory area after 60, 100 and 300 days. Depicted are mean ± SEM of n=3 (D60 and *Ccr2*$^{-/-}$ D100), n=4 (WT D300), n=5 (WT D100, *Ccr2*$^{-/-}$ D300) mice. *p=0.0396 (two-way ANOVA, Tukey's MCT).

f Histopathological subclassification of the composition of inflammatory area in WT and *Ccr2*$^{-/-}$ mice described in Supplementary Fig. 5e. Bars depict mean ± SEM.

g Representative H&E-stainings of the lungs from C57BL/6 (WT) and *Ccr2*$^{-/-}$ mice 60 and 100 days post infection on which the histopathological analysis described in Supplementary Figs. 5e and 5f was based (scale bar: 500 µm). n=3 (D60 and *Ccr2*$^{-/-}$ D100), n=5 (WT D100) mice analyzed.

h and i Bone marrow progenitors from C57BL/6 and *Ccr2*$^{-/-}$ mice were stimulated with heat-fixed BCG (10^6/ml) and M-CSF (50 ng/ml) for 6 days. MGC formation was quantified after Hemacolor staining (scale bar: 50 µm). Depicted are mean ± SEM of n=3 mice in 1 experiment, ns= not significant (p=0.0642 iMoP, p=0.9669 MC), **p=0.0067 (two-way ANOVA, Sidak's MCT).

j Bacteria per cell measured in cMoP, iMoP and MC in a killing assay approach 4 days post infection with BCG (MOI 10). Representative experiment with n=4 mice out of 2 experiments shown.

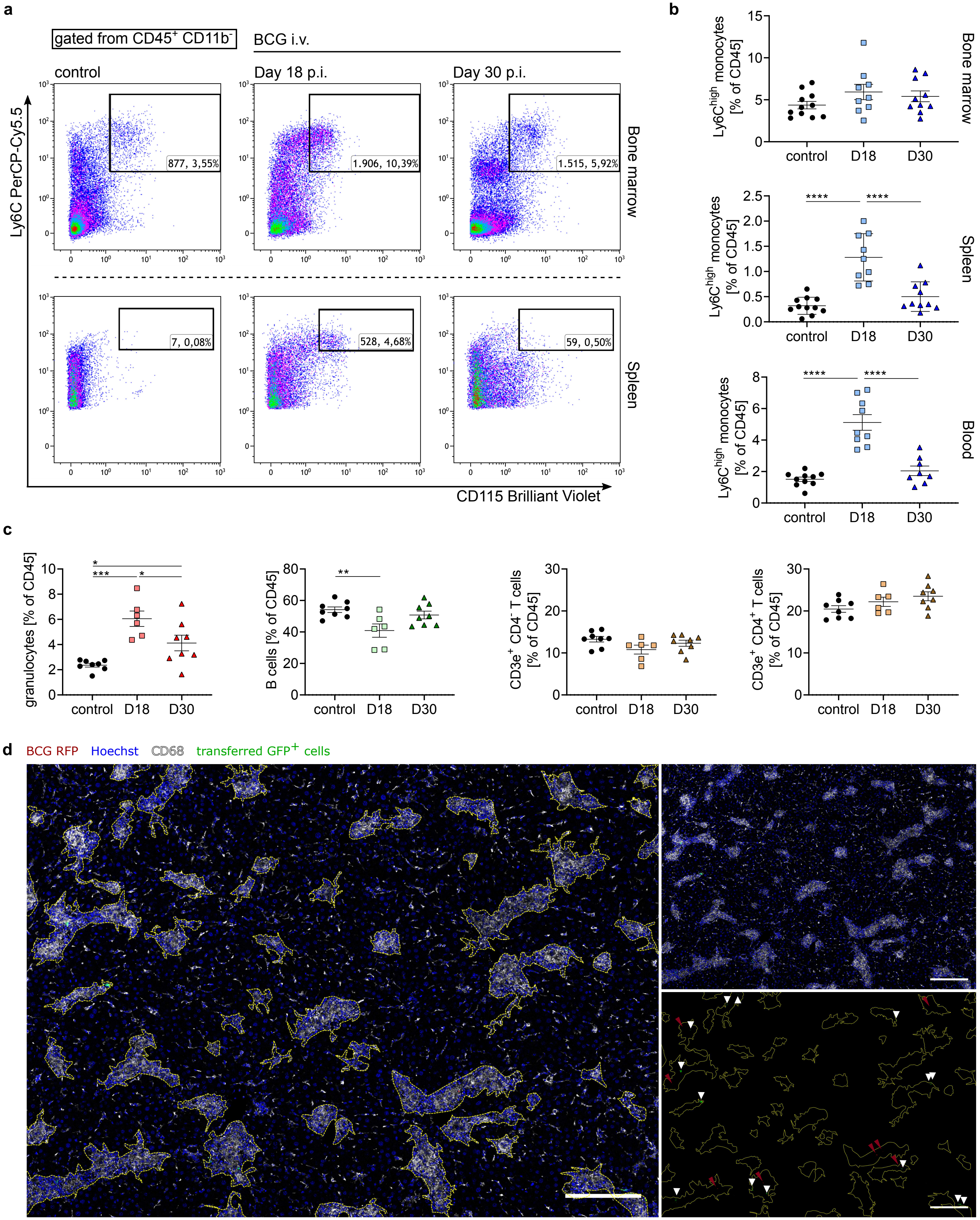
a
gated from CD45+ CD11b-
BCG i.v.
control
Day 18 p.i.
Day 30 p.i.
877, 3,55%
1.906, 10,39%
1.515, 5,92%
Bone marrow
7, 0,08%
528, 4,68%
59, 0,50%
Spleen
Ly6C PerCP-Cy5.5
CD115 Brilliant Violet
b
Ly6Chigh monocytes [% of CD45]
control
D18
D30
Bone marrow
Spleen
Blood
****
c
granulocytes [% of CD45]
B cells [% of CD45]
CD3e+ CD4- T cells [% of CD45]
CD3e+ CD4+ T cells [% of CD45]
d
BCG RFP
Hoechst
CD68
transferred GFP+ cells

Supplementary Figure 6, related to Figures 8 and 9: Alteration of further immune cell subsets in the spleen after infection with *M. bovis* BCG

a Representative dot plots of FACS analysis for Ly6C, CD115 and CD11b expression in spleen and bone marrow at day 18 and 30 post infection compared to PBS controls in described infection model (Fig. 8a).

b Frequency of Ly6C^{+} CD11b^{+} monocytes in spleen, bone marrow and blood at indicated time points compared to PBS controls in the described infection model (Fig. 8a) analyzed by flow cytometry. Depicted are mean ± SEM of n=11 (control), n=9 (D18) and n=10 (D30) mice examined over 8 experiments for spleen and bone marrow and n=10 (control), n=9 (D18) and n=8 (D30) mice examined over 7 experiments for blood. ****p< 0.0001 (one-way ANOVA, Tukey's MCT).

c Quantification of granulocytes, B-cell and T-cell subsets at indicated time points compared to PBS controls in the described infection model (Fig. 8a) analyzed by flow cytometry. Depicted are mean ± SEM of n=8 (control), n=6 (D18), n=8 (D30) mice examined over 5 experiments. *p=0.042 (control – D30), *p=0.0385 (D18 – D30), **p=0.0099, ***p=0.0002 (one-way ANOVA, Tukey's MCT).

d Overview of immunofluorescence staining of CD68-positive granulomas in the liver (yellow dotted line) after described adoptive transfer (Fig. 8g). CD68 staining is shown in white, Hoechst staining in blue and BCG-RFP in red. GFP^{+} cells (green) are highlighted with white arrows and BCG-RFP with red arrows in the image on the right. Scale bar: 200 µm. Channels were adjusted individually with respect to colour and brightness.

Supplementary Table 1: List of antibodies

Antibody	Conjugate	Clone	Manufacturer	Dilution
Anti-Ter119	Biotin	Ter119	Miltenyi Biotec	1:100
Anti-CD3e	Biotin	145-2C11	Miltenyi Biotec	1:200
Anti-CD19	Biotin	6D5	Miltenyi Biotec	1:200
Anti-SiglecF	Biotin	ES22-10D8	Miltenyi Biotec	1:100
Anti-Sca-1	Biotin	D7	Miltenyi Biotec	1:100
Anti-CD127	Biotin	A7R34 REA680	Miltenyi Biotec	1:100
Anti-CD115	PE	AFS98	Miltenyi Biotec	1:40
Anti-CD115 REAlease	PE	REAL272	Miltenyi Biotec	1:40
Anti-CD11b	VioGreen	REA592	Miltenyi Biotec	1:100
Anti-Ly6G	Biotin	1A8	BioLegend	1:200
Anti-CD117	Pacific Blue	2B8	BioLegend	1:100
Anti-CD117	BV 421	2B8	BioLegend	1:100
Anti-CD115	BV 421	AFS98	BioLegend	1:100
Anti-F4/80	BV 421	BM8	BioLegend	1:100
Anti-CD68	AF647	FA-11	BioLegend	1:100
Anti-CD16/32	purified	93	BioLegend	1:100
Anti-CD3e	APC	145-2C11	BioLegend	1:500
Isotype Rat IgG2a	BV 421	RTK2758	BioLegend	
Anti-CD4	PerCP-Cy5.5	RM4-5	eBioscience/ Thermo Fisher Scientific	1:800
Anti-CD45	eFluor 450	30-F11	eBioscience/ Thermo Fisher Scientific	1:200
Anti-CD45	PerCP Cy5.5	30-F11	eBioscience/ Thermo Fisher Scientific	1:200
Anti-CD45	FITC	30-F11	eBioscience/ Thermo Fisher Scientific	1:200
Anti-Ly6C	PerCP Cy5.5	HK1.4	eBioscience/ Thermo Fisher Scientific	1:600

Antibody	Conjugate	Clone	Manufacturer	Dilution
Anti-CD11b	APC-eFluor 780	M1/70	eBioscience/ Thermo Fisher Scientific	1:3000
Anti-F4/80	PE	BM8	eBioscience/ Thermo Fisher Scientific	1:400
Anti-CD64	PerCP-eFluor 710	X54-5/7.1	eBioscience/ Thermo Fisher Scientific	1:100
Anti-iNOS2	APC	CXNFT	eBioscience/ Thermo Fisher Scientific	FACS: 0.06 μg/test
Anti-iNOS2	PE	CXNFT	eBioscience/ Thermo Fisher Scientific	FACS: 0.06 μg/test<br>Microscopy: 1:50
Rabbit-anti-rat secondary antibody	Alexa Fluor-488		Thermo Fisher Scientific	1:100
Streptavidin	PE-Cy7		eBioscience/ Thermo Fisher Scientific	1:1000
Isotype IgG2a, κ	PE	eBR2a	eBioscience/ Thermo Fisher Scientific	
Anti-Ly6G	FITC	1A8	BD bioscience	1:200
Anti-TNFα	APC	MP6-XT22	BD bioscience	1:200
Anti-F4/80	APC	Cl:A3-1	Bio-Rad	1:100
Anti-GFP	DyLight 488	Goat polyclonal	Rockland Immunochemicals	1:1000
Anti-CCR2	purified	MC-21	Matthias Mack (Mack et al., 2001. Expression and characterization of the chemokine receptors CCR2 and CCR5 in mice. *J Immunol*. 166(7), 4697-704.)	in vitro: 1 μg/100 μl<br>in vivo: 20 μg i.p.
Isotype control	purified	MC-67		in vitro: 1 μg/100 μl<br>in vivo: 20 μg i.p.

Supplementary Table 2: Primer sequences for RT-qPCR

Gene	Forward (5' – 3')	Reverse (3' – 5')
Gapdh	ACT CCA CTC ACG GCA AAT TC	TCT CCA TGG TGG TGA AGA CA
Abca1	TGG AAA CTC ACC CAG CAA CA	GGC AGG ACA ATC TGA GCA AAG
Abcg1	CCT TCC TCA GCA TCA TGC G	CCG ATC CCA ATG TGG A
Tlr2	TTT GCT GGG CTG ACT TCT CT	AAA TCT CCA GCA GGA AAG CA
Tlr6	CCA AGA ACA AAA GCC CTG AG	TGT TTT GCA ACC GAT TGT GT
Tlr13	ATG TGA AGA CCG TGC CTT TG	GGC GGC AGA GAA AAT CCT AC
Dhcr24	GAG GGC TTG GGA TAC TGC AC	CCT TCA CAG GCC AAA TGG ATG
Fasn	GGC TCT ATG GAT TAC CCA AGC	CCA GTG TTC GTT CCT CGG A
proIL-1β	TTC AGG CAG GCA GTA TCA CTC	GAA GGT CCA CGG GAA AGA CAC
Kit	GCC TGA CGT GCA TTG ATC C	AGT GGC CTC GGC TTT TTC C
Mpo	AGG GCC GCT GAT TAT CTA CAT	CTC ACG TCC TGA TAG GCA CA
Msr1	TGG AGG AGA GAA TCG AAA GCA	CTG GAC TGA CGA AAT CAA GGA A
Itgam	ATT TTA CCG GAC TGC GTG GA	CCC ATG GCA CTC ATG GTG AT
Adgre1	ACC GTC AGG TAC GGG ATG AA	AAC TGC CAT CAA CTC ATG ATA CC
Sqle	ATA AGA AAT GCG GGG ATG TCA C	ATA TCC GAG AAG GCA GCG AAC
Hmgcr	AGA GCG AGT GCA TTA GCA AAG	GAT TGC CAT TCC ACG AGC TAT
Acat1	TGT AAA AGA CGG GCT AAC TGA TG	TGT TCC TGC CGT GAG ATA TTC AT
Acat2	CCC GTG GTC ATC GTC TCA G	GGA CAG GGC ACC ATT GAA GG
Cyp51	ATA CAA CAA TGA TCC ACA CCC C	TCA GAA CCA CAC TCT TCA ACC C

Supplementary Table 3: Number of biologically independent samples (n) for each cell type and stimulant for analysis of in vitro MGC formation (Fig. 1f)

cell type	control (-)	LM-MS (LM)	BCG MOI 20	BCG MOI 100
Lin^{-} $c\text{-}kit^{+}$	13	6	5	4
MDP	13	4	7	4
cMoP	15	6	7	4
MC	15	6	7	4

Supplementary Table 4: Number of biologically independent samples (n) used for each stimulant and time point in surface marker expression analysis (Fig. 3a and Supplementary Figs. 2a and 2b)

Stimulus	Surface marker	4h	8h	12h	48h
M-CSF (Supp. Fig. 2a)	CD64	3	3	3	3
M-CSF (Supp. Fig. 2a)	CX3CR1	7	9	8	6
M-CSF (Supp. Fig. 2a)	CD115	5	5	3	3
M-CSF (Supp. Fig. 2a)	Ly6C	4	4	4	3
M-CSF (Fig. 3a)	F4/80	4	4	3	5
M-CSF (Fig. 3a)	CD11b	7	9	8	5
M-CSF (Fig. 3a)	CD117	3	4	3 (cMoP), 4 (MC)	-
LM (Supp. Fig. 2b)	F4/80	4	4	3	5
LM (Supp. Fig. 2b)	CD11b	7	9	8	5
LM (Supp. Fig. 2b)	CD64	3	3	3	3
LM (Supp. Fig. 2b)	CD117	3	4	3 (cMoP), 4 (MC)	-

Supplementary Table 5: Number of mice (n) used for each mouse line and time point in *M.tb* aerosol infection (Figs. 6a and 6c)

	mouse line	uninfected	Day 7	Day 14	Day 21
cMoP in BM (Fig. 6a)	C57BL/6	9	5	10	10
	129S2	9	5	10	10
cMoP in blood (Fig. 6a)	C57BL/6	8	-	9	10
	129S2	9	-	9	10
iMoP in BM (Fig. 6c)	C57BL/6	9	5	10	10
	129S2	9	4	10	10
iMoP in blood (Fig. 6c)	C57BL/6	8	-	8	9
	129S2	9	-	9	10

Supplementary Table 6: Number of biologically independent samples (n) for each cell type and time point in BCG in vitro infection (Supplementary Fig. 1a)

	cell type	Day 2	Day 4	Day 6
BCG MOI 20	Lin^- $c\text{-}kit^+$	3	3	5
	MDP	5	5	7
	cMoP	5	5	7
	MC	5	5	7
BCG MOI 100	Lin^- $c\text{-}kit^+$	3	3	4
	MDP	3	3	4
	cMoP	3	3	4
	MC	3	3	4
