# Supplementary material for: Monocyte progenitors give rise to multinucleated giant cells
Source: Nat Commun. 2021 Apr 1;12:2027. doi: 10.1038/s41467-021-22103-5 (PMC8016882; doi:10.1038/s41467-021-22103-5)
Supplement: Supplementary file 3 — Reporting summary [file 41467_2021_22103_MOESM3_ESM.pdf]

nature research

Corresponding author(s): Philipp Henneke

Last updated by author(s): Jan 29, 2021

Reporting Summary

Nature Research wishes to improve the reproducibility of the work that we publish. This form provides structure for consistency and transparency in reporting. For further information on Nature Research policies, see our Editorial Policies and the Editorial Policy Checklist.

Statistics

For all statistical analyses, confirm that the following items are present in the figure legend, table legend, main text, or Methods section.

n/a	Confirmed	
☐	☒	The exact sample size (*n*) for each experimental group/condition, given as a discrete number and unit of measurement
☐	☒	A statement on whether measurements were taken from distinct samples or whether the same sample was measured repeatedly
☐	☒	The statistical test(s) used AND whether they are one- or two-sided<br>*Only common tests should be described solely by name; describe more complex techniques in the Methods section.*
☒	☐	A description of all covariates tested
☐	☒	A description of any assumptions or corrections, such as tests of normality and adjustment for multiple comparisons
☐	☒	A full description of the statistical parameters including central tendency (e.g. means) or other basic estimates (e.g. regression coefficient) AND variation (e.g. standard deviation) or associated estimates of uncertainty (e.g. confidence intervals)
☐	☒	For null hypothesis testing, the test statistic (e.g. *F*, *t*, *r*) with confidence intervals, effect sizes, degrees of freedom and *P* value noted<br>*Give P values as exact values whenever suitable.*
☒	☐	For Bayesian analysis, information on the choice of priors and Markov chain Monte Carlo settings
☒	☐	For hierarchical and complex designs, identification of the appropriate level for tests and full reporting of outcomes
☒	☐	Estimates of effect sizes (e.g. Cohen's *d*, Pearson's *r*), indicating how they were calculated

Our web collection on statistics for biologists contains articles on many of the points above.

Software and code

Policy information about availability of computer code

Data collection

•Kaluza for Gallios acquisition software, Version G 1.0, Beckman Coulter
•ZEN 2012 (blue edition), Zeiss
•FACS DIVA software (v8.0.1), Becton Dickinson
•Affymetrix GeneChip Command Console software v4.2
•Affymetrix GeneChip Command Console v4.1.3 software
•Affymetrix GeneChip Expression Console v1.4 software
- RMA algorithm [Irizarry, R. A. et al. Exploration, normalization, and summaries of high density oligonucleotide array probe level data. Biostatistics 4, 249–264 (2003).]
- GCCN-SST-RMA algorithm [http://tools.thermofisher.com/content/sfs/brochures/sst_gccn_whitepaper.pdf]
•Wave desktop software, Version 2.4.0.60, Agilent

Data analysis

•Microsoft Excel 2010
•Inkscape, Version 0.92
•Kaluza analysis software, Version 1.5, Beckman Coulter
•FlowJo analysis software, Version 10, TreeStar
•ModFit LT™, Version 4, Verity Software House
•ZEN 2012 (blue edition), Zeiss
•GraphPad Prism 7 and 8
•Software R (Version 3.2 and i386 3.3.2, R Core Team (2016). R: A language and environment for statistical computing. R Foundation for Statistical Computing, Vienna, Austria. URL https://www.R-project.org/.)
•Panther Classification System (Mi H, Muruganujan A, Casagrande JT, Thomas PD. (2013). Large-scale gene function analysis with the PANTHER classification system. Nat Protoc. 8(8), 1551-66 and Mi H, Muruganujan A, Ebert D, Huang X, Thomas PD. (2019). PANTHER

version 14: more genomes, a new PANTHER GO-slim and improvements in enrichment analysis tools. Nucleic Acids Res. 47(D1), D419-D426.)
•Software GENE-E (https://software.broadinstitute.org/GENE-E/).
•Wave desktop software, Version, 2.4.0.60 Agilent
•Imaris x64 9.5.0

For manuscripts utilizing custom algorithms or software that are central to the research but not yet described in published literature, software must be made available to editors and reviewers. We strongly encourage code deposition in a community repository (e.g. GitHub). See the Nature Research guidelines for submitting code & software for further information.

Data

Policy information about availability of data

All manuscripts must include a data availability statement. This statement should provide the following information, where applicable:
- Accession codes, unique identifiers, or web links for publicly available datasets
- A list of figures that have associated raw data
- A description of any restrictions on data availability

All microarray data are deposited at the National Center for Biotechnology Information Gene Expression Omnibus public data base. The accession numbers for the 'Genom-wide expression study of cMoP and monocytes from murine bone marrow compared to their differentiated descendants' (Fig. 3 and Supplementary Fig. 2) is GSE117456 [https://www.ncbi.nlm.nih.gov/geo/query/acc.cgi?acc=GSE117456] and for the 'Genom-wide expression study of cMoP and iMoP from murine bone marrow' (Fig. 7) is GSE117460 [https://www.ncbi.nlm.nih.gov/geo/query/acc.cgi?acc=GSE117460]. Other data supporting the results can be provided by the authors upon reasonable request.

Field-specific reporting

Please select the one below that is the best fit for your research. If you are not sure, read the appropriate sections before making your selection.

- [x] Life sciences
- [ ] Behavioural & social sciences
- [ ] Ecological, evolutionary & environmental sciences

For a reference copy of the document with all sections, see nature.com/documents/nr-reporting-summary-flat.pdf

Life sciences study design

All studies must disclose on these points even when the disclosure is negative.

Sample size	No exact statistical predetermination of sample size was performed. To fulfill the principles of the 3Rs (Replacement, Reduction and Refinement) we have chosen sample size to be sufficient to detect biological differences. For statistical analysis at least three biologically independent samples were analyzed. Numbers of samples (n) are indicated in the figure legends.
Data exclusions	Data were only excluded for technical reasons, except from Fig. 6c (iMoP in blood): Two outliers were removed for day 14 C57BL/6 (2.49) and 129S2 (8.50), calculated by Grubbs test. This is also indicated in the figure legend. We excluded one experiment in Figs. 8e/f due to high variance in the initial infection dose.
Replication	The exact number of biologically independent samples for each experiment is indicated in the figure legends or in the Supplementary Tables 3-6.
Randomization	Mice were age matched for in vivo experiments and randomly allocated to experimental groups.
Blinding	Overall the experiments were performed in a non-blinded fashion except for the histological scoring (Supplementary Figs. 5e, 5f, 5g), which was performed by a pathologist blinded to the genotype or treatment groups. The intravenous injection of BCG was performed by researchers not involved in the data acquisition and analysis. As far as applicable the acquisition and analysis of in vivo studies was performed in a semi-automated fashion (e.g. FACS) in order to minimize observer bias.

Reporting for specific materials, systems and methods

We require information from authors about some types of materials, experimental systems and methods used in many studies. Here, indicate whether each material, system or method listed is relevant to your study. If you are not sure if a list item applies to your research, read the appropriate section before selecting a response.

Materials & experimental systems

n/a	Involved in the study
☐	☒ Antibodies
☒	☐ Eukaryotic cell lines
☒	☐ Palaeontology and archaeology
☐	☒ Animals and other organisms
☒	☐ Human research participants
☒	☐ Clinical data
☒	☐ Dual use research of concern

Methods

n/a	Involved in the study
☒	☐ ChIP-seq
☐	☒ Flow cytometry
☒	☐ MRI-based neuroimaging

Antibodies

Antibodies used

Miltenyi Biotec (anti-mouse antibodies):
Ter119 Biotin, Clone: Ter119, 1:100
CD3e Biotin, Clone: 145-2C11, 1:200
CD19 Biotin, Clone: 6D5, 1:200
SiglecF Biotin, Clone: ES22-10D8, 1:100
Sca-1 Biotin, Clone: D7, 1:100
CD127 Biotin, Clone: A7R34 and REA680, 1:100
CD115 PE, Clone: AFS98, 1:40
CD115 PE REAlease, Clone: REAL272, 1:40
CD11b VioGreen, Clone: REA592, 1:100

BioLegend (anti-mouse antibodies):
Ly6G Biotin, Clone: 1A8; #127604, 1:200
CD117 Pacific Blue, Clone: 2B8; #105820, 1:100
CD117 BV 421™, Clone: 2B8; #105828, 1:100
CD115 BV 421™, Clone: AFS98; #135513, 1:100
F4/80 BV421™, Clone: BM8; #123132, 1:100
CD68 AF647, Clone: FA-11; #137004, 1:100
CD16/32 purified, Clone: 93; #101301, 1:100
CD3e APC, Clone: 145-2C11, #100312, 1:500

eBioscience/Thermo Fisher Scientific (anti-mouse antibodies):
CD45 eFluor 450, Clone: 30-F11; #48-0451-82, 1:200
CD45 PerCP Cy5.5, Clone: 30-F11; #45-0451-82, 1:200
Ly6C PerCP Cy5.5, Clone: HK1.4; #45-5932-82, 1:600
CD11b APC-eFluor 780, Clone: M1/70; #47-0112-80, 1:3000
F4/80 PE, Clone: BM8; #12-4801-80, 1:400
iNOS APC, Clone: CXNFT; #17-5920-80, 0.06 µg/100 µl
iNOS PE, Clone: CXNFT, #12-5920-80, 0.06 µg/100 µl (FACS), 1:50 (Microscopy)
CD4 PerCP Cy5.5, Clone: RM4-5; #45-0042-82, 1:800
CD45 FITC, Clone: 30-F11; #103108, 1:200

BD bioscience
Ly6G FITC, Clone: 1A8; #551460, 1:200
Anti-TNFα APC, Clone: MP6-XT22; #554420, 1:200

Bio-Rad
F4/80 APC, Clone: Cl:A3-1; #MCA497APCT, 1:100

Rockland Immunochemicals
anti-GFP DyLight 488, Goat polyclonal; #600-141-215, 1:1000

Isotype controls
Rat IgG2a Isotype PE, Clone: eBR2a; #12-4321-81 (eBioscience)
BV 421™ Rat IgG2a, κ Isotype, Clone: RTK2758; #400535 (BioLegend)

Secondary antibodies
Rabbit-anti-rat secondary antibody Alexa Fluor-488, Rat IgG; #A-21210 (Thermo Fisher Scientific), 1:100
Streptavidin PeCy7, #25-4317-82 (eBioscience), 1:1000

Provided by Matthias Mack:
anti-CCR2-Antibody (MC-21) and isotype control (MC-67). (Mack et al., 2001. Expression and characterization of the chemokine

	receptors CCR2 and CCR5 in mice. J Immunol. 166(7), 4697-704.)<br>in vitro: 1 µg/100 µl<br>in vivo: 20 µg i.p. daily
Validation	Antibodies were titrated before analysis or used according to manufacturer´s recommendations. Anti-CCR2-Antibody (MC-21) and the isotype control (MC-67) were provided by Matthias Mack. The validation was performed by Mack et al., 2001 in the cited publication. All other antibodies are from commercial sources. Validation data were provided in the data sheets by the manufacturers (Miltenyi Biotec, BioLegend, eBioscience/Thermo Fisher Scientific, BD bioscience, Rockland Immunochemicals, Bio-Rad).

Animals and other organisms

Policy information about studies involving animals; ARRIVE guidelines recommended for reporting animal research

Laboratory animals	All knock-out mice were on C57BL/6 genetic background and used at 6 to 10 weeks of age. Mice of both sex were included, if not otherwise indicated. C57BL/6J and C57BL/6N mice were purchased from Jackson Laboratories (USA) or Charles River Laboratories (Germany). Cx3cr1 gfp/+ mice were obtained as a kind gift from Steffen Jung (Weizmann Institute, Israel). iNOS-deficient mice (Nos2tm1Lau) and β-actin-gfp/+ (C57BL/6-Tg(CAGEGFP) 131Osb/LeySopJ) were purchased from Jackson Laboratories (USA). Andreas Diefenbach (Institute of Microbiology, Infectious Diseases and Immunology, Berlin) provided CCR2-deficient mice as a kind gift. CCR2-deficient mice for M.tb infection were purchased from Jackson Laboratories (USA). Mice were bred in animal facilities of the University of Freiburg under specific pathogen-free conditions. For M.tb infections 129S2 and C57BL/6J mice, originally bought from Charles River Laboratories (Germany), were bred at the Max-Planck-Institute for Infection Biology in Berlin. Mice between 9 and 12 weeks old were infected and maintained under Biosafety level 3. IL-13tg mice were on a C57BL/6 genetic background and bred under specific pathogen-free conditions at the Research Centre Borstel.
Wild animals	The study did not involve wild animals.
Field-collected samples	The study did not involve field-collected samples.
Ethics oversight	Animal experiments under Biosafety Level 2 were approved by the Regierungspräsidium Freiburg (G-19/171). Animal experiments under Biosafety Level 3 at the MPI in Berlin were approved by the State Office for Health and Social Services (Landesamt für Gesundheit und Soziales), Berlin, Germany (G040393/12). Infection of IL-13tg mice with M.tb were performed under Biosafety level 3 and approved by the Animal Research Ethics Board of the Ministry of Energy, Agriculture, the Environment, Nature and Digitalization Schleswig-Holstein, Kiel, Germany (approval number 3-1/19). The M.tb infection experiments of CCR2-deficient mice were in accordance with the Washington University in St. Louis Institutional Animal Care and Use Committee guidelines.

Note that full information on the approval of the study protocol must also be provided in the manuscript.

Flow Cytometry

Plots

Confirm that:

- [x] The axis labels state the marker and fluorochrome used (e.g. CD4-FITC).
- [x] The axis scales are clearly visible. Include numbers along axes only for bottom left plot of group (a 'group' is an analysis of identical markers).
- [x] All plots are contour plots with outliers or pseudocolor plots.
- [x] A numerical value for number of cells or percentage (with statistics) is provided.

Methodology

Sample preparation	For isolation of blood monocytes mice were anaesthetized with Ketamin/Xylazin i.p. and blood was drawn from the retro-orbital plexus or the inferior vena cava. Erythrocytes were lyzed in RBC Lysis buffer solution (eBioscience). Spleen cells were smashed through a 70 µm cell strainer and liver cells through a 100 µm cell strainer. Cell suspensions were pelleted and erythrocytes were lyzed in RBC Lysis buffer solution (eBioscience). Bone marrow was collected by flushing femur and tibia. Cells were passed through a 70 µm cell strainer. Prior to further antibody staining cells were washed in FACS buffer (1 % FBS, 2 mM EDTA). Further details about sample preparations for individual assays are provided in the Methods section.
Instrument	Flow cytometers: BD LSR Fortessa flow cytometer, Becton Dickinson; Gallios™ flow cytometer, Becton Dickinson; CytoFLEX S, Becton Dickinson<br>Fluorescence-activated cell sorting: MoFlo® Astrios™ cell sorter, Beckman Coulter; FACS Aria™ Illu, BD Bioscience
Software	Acquisition:<br>• Kaluza for Gallios acquisition software, Version G 1.0, Beckman Coulter<br>• FACS DIVA software (v8.0.1), Becton Dickinson<br>Analysis:<br>• Kaluza analysis software, Version 1.5, Beckman Coulter<br>• FlowJo analysis software, Version 10, TreeStar
Cell population abundance	Fluorescence-activated cell sorting was performed in our Core Facility (Lighthouse Fluorescence Technologies Core Facility, Medical Center, University of Freiburg) and in the Core Facility of the Research Centre Borstel with technical assistance from

FACS operators. The protocol set-up was determined as described below. To reach a high purity of the cell populations we combined magnetic cell separation with fluorescence-activated cell sorting. Apart from this a strict gating strategy was applied in order to exclude intermediate 'positive' or 'negative' cell populations and receive pure cell populations. Post-sort analysis was performed occasionally (see Fig. 3a). The gating strategy was controlled regularly.

Gating strategy

In general live cells were gated by FSC-A/SSC-A, if necessary doublets were excluded in a FSC-W/SSC-W plot. The gating strategy for isolation of the different progenitor subsets is provided in Figs. 1c, 6b and Supplementary Fig. 5b. Progenitor gating in Figs. 6a/c/g was additonally combined with a CD45 and a live/dead staining. Gate boundaries were defined by FMO stainings. Selected stainings were controlled with isotype controls. Positive or negative controls were applied whenever possible (Apoptosis staining, TNFα staining).

[x] Tick this box to confirm that a figure exemplifying the gating strategy is provided in the Supplementary Information.
